# Inpatient Rehabilitation Psychiatry: A Literature Review and Illustrative Case Series

**DOI:** 10.1192/j.eurpsy.2025.826

**Published:** 2025-08-26

**Authors:** A. Keng, G. D. Eom, O. Ghaffar

**Affiliations:** 1 Psychiatry, Baycrest Centre for Geriatric Care; 2 Psychiatry, University of Toronto; 3 Psychiatry, Sinai Health, Toronto, Canada

## Abstract

**Introduction:**

Physical rehabilitation is usually required after serious illness or injury; there is a significant need for simultaneous psychiatric rehabilitation as well. Psychiatric consultation to inpatient rehabilitation units presents with unique clinical questions distinct from consultation to other units.

**Objectives:**

To present a literature review and an illustrative case series of common psychiatric referrals from physical rehabilitation units.

**Methods:**

First, the current literature was reviewed on PubMed looking at current models of psychiatric consultation to inpatient physical medicine and rehabilitation units. Second, cases were reviewed from tertiary post-acute care units including stroke, acquired brain injury, post-fracture fast and slow stream, and complex continuing care. Third, thematic analysis was performed to extract illustrative themes presented in a case series of four mock patients.

**Results:**

There is a paucity of literature on psychiatric consultation to inpatient physical rehabilitation units. The current literature, however, supports high utilization of psychiatric consultation in comorbid physical illness (Daly et al 2023, PMR). Four major themes emerged on psychiatric consultation to inpatient physical rehabilitation: (1) Interdisciplinary management of basophobia [the fear of falling], (2) Managing severe persisting mental illness and addressing medications that increase the risk of falling, (3) Managing new onset trauma-related disorders and pain-related disorders, (4) Assessing partial cognitive improvement and impairment in the setting of brain injury.

**Conclusions:**

Psychiatrists often deal with patients recovering from an episode of serious physical illness or injury. There is significant overlap between the work done in physical and psychiatric rehabilitation. Patient and staff education, judicious prescribing and de-prescribing, and interdisciplinary management are critical to successful outcomes.

**Disclosure of Interest:**

None Declared

